# A Novel Controller Design for the Next Generation Space Electrostatic Accelerometer Based on Disturbance Observation and Rejection

**DOI:** 10.3390/s17010021

**Published:** 2016-12-23

**Authors:** Hongyin Li, Yanzheng Bai, Ming Hu, Yingxin Luo, Zebing Zhou

**Affiliations:** 1 MOE Key Laboratory of Fundamental Quantities Measurement, School of Physics, Huazhong University of Science and Technology (HUST), Wuhan 430074, China; hongyin83li@hust.edu.cn (H.L.); abai@mail.hust.edu.cn (Y.B); 2 School of Automation, Huazhong University of Science and Technology (HUST), Wuhan 430074, China; 3 Institute of Geodesy and Geophysics, Chinese Academy of Science, Wuhan 430077, China; huming@whigg.ac.cn; 4 Tianqin Research Center for Gravitational Physics, School of Physics and Astronomy, Sun Yat-sen University, Zhuhai 519082, China; luoyx43@mail.sysu.edu.cn

**Keywords:** electrostatic accelerometer, inertial sensor, embedded model control, non-smooth optimization

## Abstract

The state-of-the-art accelerometer technology has been widely applied in space missions. The performance of the next generation accelerometer in future geodesic satellites is pushed to $8\times 10^{-13}\;m/s^{2}/Hz^{1/2}$, which is close to the hardware fundamental limit. According to the instrument noise budget, the geodesic test mass must be kept in the center of the accelerometer within the bounds of 56 $\mathrm{pm}/{\mathrm{Hz}}^{1/2}$ by the feedback controller. The unprecedented control requirements and necessity for the integration of calibration functions calls for a new type of control scheme with more flexibility and robustness. A novel digital controller design for the next generation electrostatic accelerometers based on disturbance observation and rejection with the well-studied Embedded Model Control (EMC) methodology is presented. The parameters are optimized automatically using a non-smooth optimization toolbox and setting a weighted H-infinity norm as the target. The precise frequency performance requirement of the accelerometer is well met during the batch auto-tuning, and a series of controllers for multiple working modes is generated. Simulation results show that the novel controller could obtain not only better disturbance rejection performance than the traditional Proportional Integral Derivative (PID) controllers, but also new instrument functions, including: easier tuning procedure, separation of measurement and control bandwidth and smooth control parameter switching.

## 1. Introduction

High Precision Electrostatic Space Accelerometers (HPESAs) are the spaceborne measuring instruments of non-conservative accelerations of space vehicles. They had been and are the main payloads of geodetic satellites, such as the Challenging Minisatellite Payload (CHAMP), the Gravity Recovery, Climate Experiment (GRACE), the Gravity Field and Steady State Ocean Circulation Explorer (GOCE) [1], the GRACE follow-on [2] and space fundamental physics [3,4]. The next generation satellite to satellite tracking mission (GRACE-type) and gradiometer mission (GOCE-type) require performances of their HPESAs to be able to achieve the levels of 10${}^{-11}$ $m/s^{2}/{\mathrm{Hz}}^{1/2}$ [5] and $8\times 10^{-13}$ $m/s^{2}/{\mathrm{Hz}}^{1/2}$ [6], respectively. Moreover, spaceborne gravitational wave detectors, such as the Laser Interferometer Space Antenna (LISA) [7] and the TianQin mission of China [8], need inertial-sensor type HPESAs to play the role of geodesic references in the center of satellites. The TianQin’s accelerometers share the same principle of the capacitance sensor and electrostatic actuator with the one in geodetic missions, but have higher acceleration measurement precision of up to $10^{-15}$ $m/s^{2}/{\mathrm{Hz}}^{1/2}$. All of these state-of-the-art HPESAs, called the next generation electrostatic accelerometers, have noises close to the physical fundamental limits and, thus, call for novel designs of their feedback controllers.

**Figure 1:**
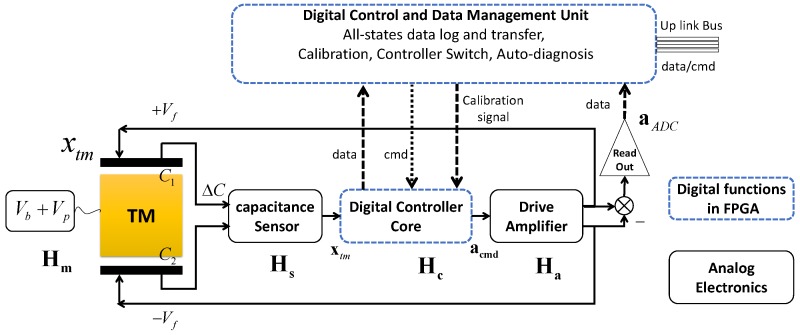
Schematic block-diagram of a High Precision Electrostatic Space Accelerometer (HPESA) in one dimension; TM, Test Mass; H, transfer functions of different components; $V_{f}$, feedback Voltage; $a_{ADC}$, acceleration output by the ADC read out; cmd means controller commands.

A typical HPESA consists of a Test Mass (TM) and three surrounding pairs of electrodes to form the differential capacitances for both the position sensors [9,10,11] and the electrostatic actuators that balance the input acceleration. The simplified block-diagram of an HPESA is shown in Figure 1. The actuation voltage commands are generated by a feedback controller, which is driven by the TM position measurements. As the core of instrument electronics, the controller is in charge of stabilizing the TM within a small range about the sensor cage center, also known as the equilibrium point, where the accelerometer attains the performance decided by the hardware noise floor. When the TM stays within this range, the nonlinearities of both the position sensor and actuation force are maintained at a negligible level, so that the feedback read out can accurately and linearly measure the accelerations applied to the accelerometer frame [12].

The ultra-high resolution of the next generation electrostatic accelerometer could only be achieved after the on-orbit calibration of the scaling factor, bias and coupling factors, etc. Full-time monitoring of the working states is an essential function for instrument diagnosis and scientific measurement, which demands all motion information output of the TM. In this case, a “Digital Control and Data Management Unit” (DCDMU) in Figure 1, which should have function modules including all state data log/transfer, controller switching for different working modes, as well as the in-orbit diagnosis/calibration, is designed for the next generation of HPESA. All of these functions indicate that the digital controller should be in a state observer-based form. The state observer in controllers will filter and output the TM motion states in real time and is the core block connecting these functions with the feedback servo of TM seamlessly, as shown in Figure 1. In this figure, DCDMU is composed of a Digital Controller Core (DCC) in the feedback loop and a management unit, which are connected by three signal routes: data, cmdand calibration signal. This three routes are respectively responsible for scientific data logging and transfer, working mode switching command and calibration signal injection. The DCC and the whole DCDMU will be deployed in the same FPGA chip, and their detailed structures will be described in the following sections. This study was carried out to improve the performance and functions of the next generation Chinese HPESA built in HUST (Huazhong University of Science and Technology, China), whose flight model has been launched in 2013 [10].

Traditional Proportional Integral Derivative (PID) controllers have been applied to the accelerometers of GRACE and the GRACE follow-on, as well as the GOCE’s electrostatic gravity gradiometer [13], but their control algorithms are not systematically reported in the literature. Modern control theories, such as Kalman filter-based controllers and H-infinity controllers, have been adopted in many precision space missions, such as the attitude control system [14] of the Microsatellite mission for the test of the equivalence principle [15,16] and the drag-free control system of the LISA-Pathfinder [17]. The Kalman filter is based on the least-square procedure of states of the object variables in the time domain, but lacks a targeted design on full information about signal/noise spectral characteristics. On the other hand, H-infinity technology, which could be defined as a theory based on infinity norm in Hardy Space, guarantees an accurate design of the spectral characteristics of the controller transfer functions, but usually ends in complex high-order controllers.

Embedded Model Control (EMC) [18], which makes use of an extended disturbance observation and rejection scheme, has been well studied in the motion control system and has shown a great potential in precise frequency domain design. However, before tuning of EMC, equations describing the relations between the closed-loop poles’ location and dynamic feedback gains need to be derived case by case in the previous procedure. Therefore, rich experiences are required in the determination of the feedback gains in order to achieve the frequency performance goal, which is too complicated for ordinary users. In this work, a non-smooth optimization method based on numerical tools has been developed to avoid the complicated derivation process in EMC and has shown the abilities to directly design the closed-loop transfer function of controllers in various cases with a common program architecture. Moreover, this work also combines the state observer-based architecture of EMC with the H-infinity technology and, thus, inherits both the advantages of the optimized operations of physical states in the time domain and accurate frequency domain performances. In this paper, following the EMC guidelines, a state observer and the whole DCC have been designed based on an HPESA discrete time state space model in Section 2, Section 3 and Section 4. The method of using non-smooth optimization for tuning the loop shape of the whole system is described in Section 5. In Section 6, the designed and tuned controller has been simulated with an end-to-end numerical model of the HPESA. The accuracy of the loop shape design and the flexibility of the design algorithm are pointed out. Conclusions of the work are presented in Section 7. The nominal parameters of an HPESA from HUST designed for the future Chinese gradiometer mission are listed in Table 1 as our study case.

## 2. Control Design Requirement

The relative dynamics of the TM can be described by the relation of ${\ddot{x}}_{\mathrm{TM}}=-a_{in}+a_{ele}$, where $x_{\mathrm{TM}}$ is the relative position of the TM. When the command acceleration $a_{ele}$ precisely cancels the input acceleration $a_{in}$, ${\ddot{x}}_{\mathrm{TM}}$ becomes negligible, so then $a_{ele}=a_{in}$. The relation between $a_{ele}$ and the control voltage $V_{f}$ is given by [19]:

(1) $$a_{ele}=\frac{2C_{0}}{m_{\mathrm{TM}}d_{0}}\left[-V_{b}V_{f}+\frac{\left(V_{b}^{2}+V_{prms}^{2}\right)x_{\mathrm{TM}}}{d_{0}}+\frac{V_{f}^{2}x_{\mathrm{TM}}}{d_{0}}\right],$$

where $C_{0}$, $m_{\mathrm{TM}}$, $d_{0}$, $V_{b}$ and $V_{prms}$ represent, respectively, balance capacitance, TM mass, balance gap, bias voltage and the RMS of the modulation voltage. Their typical values are listed in Table 1. Equation (1) can be rewritten as:

(2) $$a_{ele}=H_{a,dc}V_{f}+\omega_{e}^{2}x_{\mathrm{TM}}+\frac{\omega_{e}^{2}x_{\mathrm{TM}}V_{f}^{2}}{V_{b}^{2}+V_{prms}^{2}},$$

with definitions of $H_{a,dc}=-2C_{0}V_{b}/m_{\mathrm{TM}}d_{0}$ as the scale factor and $\omega_{e}^{2}=2C_{0}\left(V_{b}^{2}+V_{prms}^{2}\right)/m_{\mathrm{TM}}d_{0}^{2}$ as the electrostatic stiffness. In Equation (2), the first term on the right-hand side is the nominal feedback acceleration $a_{ele,norm}$, which is proportional to the feedback voltage $V_{f}$; the second and third terms are respectively the linear stiffness coupling and the cubic acceleration term, which must be suppressed to a level below the acceleration noise floor $a_{req}$ given by:

(3) $$a_{ele,aff}=\omega_{e}^{2}x_{\mathrm{TM}}\left(1+\frac{V_{f}^{2}}{V_{b}^{2}+V_{prms}^{2}}\right)<a_{req}.$$

In this way, the performance of the feedback controller in terms of $x_{\mathrm{TM}}$ must fulfill the following inequality in the Measurement Bandwidth (MBW):

(4) $$x_{\mathrm{TM},req}<\alpha \omega_{e}^{-2}{\left(1+\frac{V_{f\max}^{2}}{V_{b}^{2}+V_{prms}^{2}}\right)}^{-1}a_{req},$$

where α=0.5 is set as the redundancy factor and $V_{f\max}$ is the maximal feedback voltage with its typical value listed in Table 1. Here, the redundancy factor *α* is used during the noise budget to leave enough margins for other noise sources that are not from the control system.

The acceleration noise floor $a_{req}$ of a next generation HPESA for the gradiometer mission is defined by a piecewise spectral curve with a flat level of $8\times 10^{-13}$ $m/s^{2}/{\mathrm{Hz}}^{1/2}$ in the measurement band from 5 mHz–100 mHz, a decreasing slope of −20 dB/dec below 5 mHz and an increasing slope of 40 dB/dec above 100 mHz. This increasing slope is caused by the second derivative of the flat noises of the position sensor: $x_{n}\omega^{2}$ (with $x_{n}$ defined in Table 1), which is not repeated in the definition of the $x_{\mathrm{TM}}$ requirement. Using Equation (4) and the parameters of Table 1, the requirement of $x_{\mathrm{TM},req}<5.73$ $\mathrm{pm}/{\mathrm{Hz}}^{1/2}$ can be fixed in the mid-frequency region of the acceleration noise (5 mHz–100 mHz) and continues the flat constant above 100 mHz. Below this region (<5 mHz), its spectral bound follows the −20 dB/dec slope of $a_{req}$.

## 3. Model of The Electrostatic Accelerometer

The TM of the HPESA is a precisely-machined cubic titanium coated with gold. Stringent requirements of the squareness and parallelism between surfaces guarantee negligible cross-coupling coefficients between the three measurement axes. The accelerator control plant can be decomposed into three decoupled single-input single-output (SISO) dynamic systems. The transfer function of each SISO system can be subdivided into three main transfer functions, as illustrated in Figure 1. In the figure, $H_{m}\left(s\right)$ is the transfer function of the TM motion dynamics in the mechanical sensor head; $H_{s}\left(s\right)$ and $H_{a}\left(s\right)$ are the transfer functions of the position sensor and of the actuator, respectively. Using $H_{m}$, $H_{s}$ and $H_{a}$, the transfer function of the controller can be designed on the basis of the closed-loop requirements.

Since the bandwidth of $H_{s}\left(s\right)$ and $H_{a}\left(s\right)$ is close to 300 Hz, which is far above the 10-Hz sampling rate of the accelerometer scientific data [20], they are approximated by static and unit gains, so that $H_{s}\left(s\right)\approx 1$ and $H_{a}\left(s\right)\approx 1$. Then, the whole actuator-to-sensor dynamics M can be approximated as equal to the TM motion dynamics: $M=H_{a}H_{m}H_{s}\approx H_{m}$. The correct discrete-time model of $H_{m}$ is essential for the controller design. The relative motion dynamics of TM inside the capacitance cage, which is accounted by $H_{m}$, is given by:

(5) $${\ddot{x}}_{\mathrm{TM}}=\omega_{e}^{2}x_{\mathrm{TM}}+\left(-a_{in}+a_{fed}\right),$$

where $a_{in}$ is the sum of all input accelerations to be measured and $a_{fed}$ represents the feedback acceleration commanded by the actuator. Equation (5) can be expressed in a discrete time state equation as follows:

(6) $$\begin{array}{c} x_{c}\left(i+1\right)=A_{c}x_{c}\left(i\right)+B_{c}u\left(i\right)+B_{d}d\left(i\right)\\ y_{m}=C_{c}x_{c}\left(i\right)\\ x_{c}={\left[\begin{array}{cc} x_{\mathrm{TM}} & v_{\mathrm{TM}} \end{array}\right]}^{T},u=a_{fed},d=a_{in},y_{m}=y_{m}\\ A_{c}=\left[\begin{array}{cc} 1 & T_{s}\\ \omega_{e}^{2}T_{s} & 1 \end{array}\right],B_{d}=B_{c}=\left[\begin{array}{c} 0\\ T_{s} \end{array}\right],C_{c}=\left[\begin{array}{cc} 1 & 0 \end{array}\right]. \end{array}$$

In this plant, $v_{\mathrm{TM}}$ in the plant state vector $x_{c}$ is the relative speed of the TM, $T_{s}$ is the sampling time of the digital control loop, u is the control command input and $y_{m}$ is the model output corresponding to the position of the TM. $A_{c}$, $B_{c}$, $B_{d}$ and $C_{c}$ are the state matrix, control input matrix, disturbance input matrix and measurement output matrix, respectively. Here, d is the acceleration disturbance to be measured and should be eliminated completely. Accurate estimation of d could be directly used as the measurement output, and its elimination is the main scope of this control system. Detailed modeling of d will be discussed in the following section (Section 4.2). Equation (6) will be used as the embedded model of the controllable dynamics of the plant in the next section. The transfer functions $H_{s}$ and $H_{a}$ are treated as neglected dynamics during the controller design while explicitly included in the fine numerical simulation model of the whole system. The spectral densities of the position sensor noise and of the actuator noise are given in Table 1.

## 4. EMC Design for the Accelerometer

### 4.1. Background of EMC

During modeling of the plant and its environment, input disturbances can be described as linear systems driven by random processes. Proper forms of connection and structure could form an all states observable model, based on which the environment disturbance can be observed and compensated. This inspires several different control theories called the “disturbance rejection controller” [21,22], being widely studied and applied to many fields. EMC as one of the “disturbance rejection” methodologies was founded on the discrete time state space theory with direct deployment convenience in the computer. The theory of EMC was outlined in [18] and had been applied to different cases, including the early design of the drag-free and attitude control of the GOCE satellite [23,24]. The relation between the predictor loop and the whole control loop [25] will lead us to a practical design procedure.

**Figure 2:**
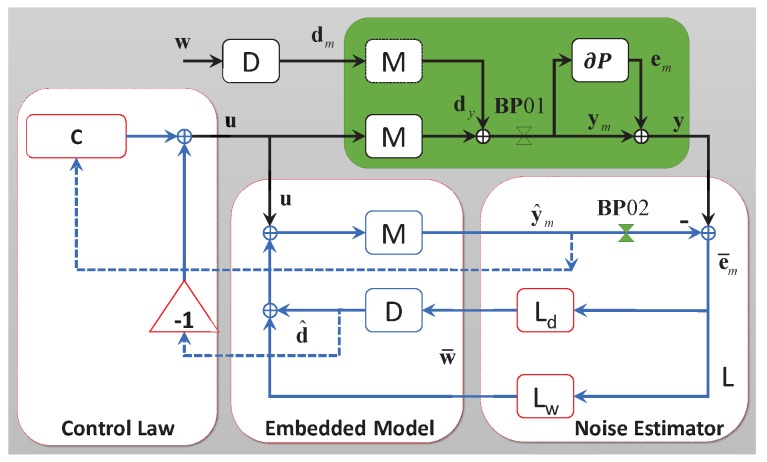
General block-diagram of the Embedded Model Control (EMC); M, plant dynamics; D, disturbance dynamics; V, noise estimator; C, controller; **BP**, Break Point.

The controller design of the accelerometer is a typical motion control problem, which may help us in finding clear physical meanings of each EMC concept. The fundamental idea of EMC is that the DCC should contain a “copy” of the real controlled plant and of its disturbance environment (the so-called embedded model), as seen in Figure 2. The embedded model is the core of the EMC and is surrounded by the noise estimator L on the right side, as well as the control law on the left side. It propagates the plant state $x_{c}$ and the disturbance state $x_{d}$ to get a full state prediction $\hat{x}\left(i+1\right)={\left[{\hat{x}}_{c}\left(i+1\right)\;{\hat{x}}_{d}\left(i+1\right)\right]}^{T}$.

### 4.2. Building EMC Structure for the Accelerometer

**Figure 3:**
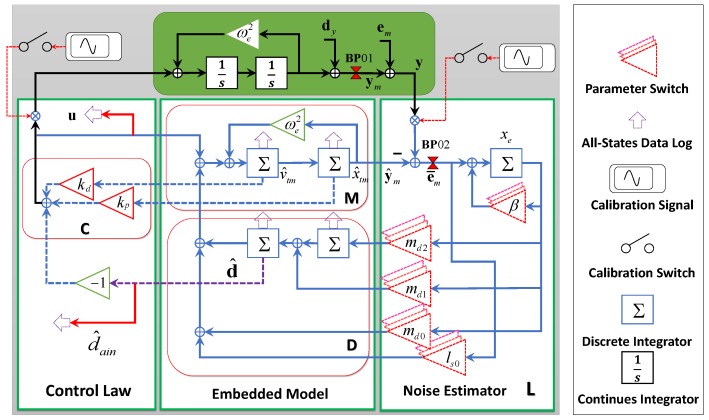
Detailed block-diagram of the EMC-based digital controller of the HUSTaccelerometer and its relations with the Digital Control and Data Management Unit (DCDMU).

The detailed block-diagram of the electrostatic accelerometer embedded model in Figure 3 is obtained by expanding each block of Figure 2. The embedded model consists of the plant dynamics M given by Equation (6) and of the disturbance dynamics D. Their state vectors are $x_{c}$ and $x_{d}$, respectively. The disturbance state $x_{d}$ is synthesized by a cascade of Discrete Time (DT) integrators, driven by a white noise vector w with bounded covariance. The main input disturbances of the accelerometer—the input acceleration to be measured and the actuator noise—can be modeled as a second-order dynamics, which is shown as the block D in Figure 3. A second-order dynamics is selected because it could describe a random disturbance with the slope of Power Spectral Density (PSD) of up to −40 dB/dec, while in this work, the input acceleration is between −20 dB/dec and −40 dB/dec (will be further discussed in Section 6). During modeling, the disturbance source is assumed to be in the form of acceleration without additional velocity disturbance, which is the actual situation of the relative motion dynamics of TM. In this case, the output of the disturbance dynamics enters the controllable dynamics M from the same actuator injection point, which is called a “collocated” situation. However, this does not always hold for other cases. Considerations of the non-collocated situations have been deeply studied in [18]. In Figure 3, each Σ block represents a DT integrator $T_{s}{\left(z-1\right)}^{-1}$, where $z=e^{-sT_{s}}$. The disturbance state equation of D is given by:

(7) $$\begin{array}{c} x_{d}\left(i+1\right)=A_{d}x_{d}\left(i\right)+G_{d}w\left(i\right),\\ d\left(i\right)=E_{d}x_{d}\left(i\right)+E_{w}w\left(i\right),\\ A_{d}=\left[\begin{array}{cc} 1 & T_{s}\\ 0 & 1 \end{array}\right],G_{d}=\left[\begin{array}{ccc} 0 & T_{s} & 0\\ 0 & 0 & T_{s} \end{array}\right],E_{d}=\left[\begin{array}{cc} 1 & 0 \end{array}\right],\\ E_{w}=\left[\begin{array}{ccc} 1 & 0 & 0 \end{array}\right], \end{array}$$

where $A_{d}$, $G_{d}$, $E_{d}$ and $E_{w}$ are the state matrix, input matrix, output matrix and feedforward matrix, respectively.

The noise estimator L is the feedback dynamics driving the embedded model state vector $\hat{x}$ to asymptotically converge to “true” state x using the model error ${\bar{e}}_{m}=y-{\hat{y}}_{m}$, with y and ${\hat{y}}_{m}$ as the measured and the model-estimated TM positions, respectively. During the predictor loop design of EMC, only noise channels of D have feedbacks to stabilize the loop. The absence of the velocity state disturbance channel leads to only three tunable gains ($m_{d0}$, $m_{d1}$, $m_{d2}$) for four independent states ($x_{d1}$, $x_{d2}$, $v_{tm}$, $x_{tm}$) when only static feedback gains are considered in L. This will cause the system to be uncontrollable. In order to guarantee the controllability of the predictor loop, an additional state $x_{e}$ is inserted into L. It turns L from a static feedback as in classical state predictors into a first-order dynamic feedback. The state $x_{e}$ is described in a first-order dynamics with a tunable parameter *β* as follows:

(8) $$x_{e}\left(i+1\right)=\left(1-\beta \right)x_{e}\left(i\right)+\left(y\left(i\right)-{\hat{y}}_{m}\left(i\right)\right),$$

and by using the transfer function form of the first-order dynamics in Equation (8): ${\left(\left(z-1\right)+\beta \right)}^{-1}$, with the tunable gains $m_{d}$ and $l_{s}$, L becomes:

(9) $$L=m_{d}{\left(z-1+\beta \right)}^{-1}+l_{s},$$

where $m_{d}={\left[\begin{array}{ccc} m_{d0} & m_{d1} & m_{d2} \end{array}\right]}^{T}$ and $l_{s}={\left[\begin{array}{ccc} l_{s0} & 0 & 0 \end{array}\right]}^{T}$, as shown in the block L of Figure 3. $L_{d}$ and $L_{w}$ in Figure 2 are described, respectively, by the first two rows and the last row of L.

In the control law block of Figure 3, the predicted state feedback and the disturbance prediction feedforward together force the plant state to the target trajectory using the command $u\left(i\right)$. This command includes the disturbance rejection $\hat{d}\left(i\right)=E_{d}{\hat{x}}_{d}\left(i\right)$ and the state feedback $K_{c}\left[{\hat{x}}_{c}\left(i\right)-x_{targ}\left(i\right)\right]$, which drives the plant state to converge to the target $x_{targ}$. $x_{targ}$ is usually produced by a module called the reference generator [18] to guide the plant state maneuver smoothly. In our preliminary design set-up, the TM is well protected by 16 position stop rods, where the overshoot of TM position is tolerable. Therefore, the target is set to zero position and zero velocity in the capacitance cage in our design for simplicity purposes. $u\left(i\right)$ can then be expressed as:

(10) $$u\left(i\right)=-\hat{d}\left(i\right)+K_{c}{\hat{x}}_{c}\left(i\right),$$

where $K_{c}{\hat{x}}_{c}$ is equivalent to a Proportional Derivative (PD) controller, and by taking the embedded model output ${\hat{y}}_{m}$ as the control law input, it is rewritten as:

(11) $$\begin{array}{c} K_{c}{\hat{x}}_{c}=\left[\begin{array}{cc} k_{p} & k_{d} \end{array}\right]\left[\begin{array}{c} {\hat{x}}_{TM}\\ {\hat{v}}_{TM} \end{array}\right]\\ =\left(k_{p}+k_{d}T_{s}^{-1}\left(z-1\right)\right){\hat{y}}_{m}=C\left(z\right){\hat{y}}_{m}. \end{array}$$

In this equation, only the PD control law is presented, because in EMC, the low-frequency acceleration disturbance is already estimated by the disturbance predictor and fed into the control law to cancel the static errors. This rejection part in EMC can be regarded as a double integrator. For an inertial sensor controller whose TM has a bigger free space and no protection stop rod, a specific reference generator should be included in the future.

In Figure 3, the controller working mode could be switched by changing five gain parameters in the noise estimator. All of the estimated states are registered in the discrete integrator during switching, which will guarantee a smooth feedback control. Data to be logged are marked by purple arrows, including all of the observed states and output commands. Calibration signals and injection points are also marked in the figure.

### 4.3. Transfer Function Design of EMC

The embedded model, which consists of M, D and of the noise estimator L, constitutes the state predictor of the plant and disturbance. It is called the “state predictor”, since it forecasts the state at the next time step using the current state and input values of each DT integrator (Σ block). The loop from the M output port back to the M input port, passing through L and D is the Predictor Loop (PL). The main input and output of the PL are the sensor measurement y, the predicted output ${\hat{y}}_{m}$ and the prediction error ${\hat{e}}_{m}=y_{m}-{\hat{y}}_{m}$. The performance of the state predictor is mainly described by ${\hat{e}}_{m}$, whose relation to the model error $e_{m}=y-y_{m}$ and to the equivalent disturbance $d_{y}$ according to the EMC theory [18] is:

(12) $${\hat{e}}_{m}=-V_{m}e_{m}+S_{m}d_{y}.$$

The transfer function $V_{m}$ from $e_{m}$ to ${\hat{e}}_{m}$ is the predictor complementary sensitivity. From Figure 3, $V_{m}$ is the transfer function from y to ${\hat{y}}_{m}$, revealing the confidence of the predictor (output ${\hat{y}}_{m}$) to the measurement (input y). The wider the bandwidth of $V_{m}$, the more information/noise of y will be repeated in ${\hat{y}}_{m}$. Similarly, the transfer function from $d_{y}$ to ${\hat{e}}_{m}$ is the sensitivity $S_{m}$, which is a high-pass filter rejecting the outside disturbance in the stopband. Both transfer functions can be derived from Equations (6)–(9) in the form of the state space [18]. For simplicity purposes, $V_{m}$ and $S_{m}$ are given as follows:

(13) $$\begin{array}{c} S_{m}={\left(1+U_{m}\right)}^{-1}\\ V_{m}=1-S_{m}=U_{m}{\left(1+U_{m}\right)}^{-1} \end{array},$$

where $U_{m}=\mathrm{MDL}$ is the open-loop transfer function of the state predictor.

The EMC design criterion is similar to other Linear Time-Invariant (LTI) controllers: (1) shaping of the sensitivity function S of the Whole Control Loop (WCL) in order to fulfill the requirement of low-frequency disturbance rejection; (2) shaping of the complementary sensitivity function V in order to meet the requirements in the high-frequency band for sensor noise and neglected dynamics rejection. Similar to the state predictor transfer functions, $S_{m}$ and $V_{m}$, S and V are derived by investigating input and output variables at the break point BP01 in Figure 3, which provides:

(14) $$S\left(z\right)=y\left(z\right)/e_{m}\left(z\right),V\left(z\right)=y_{m}\left(z\right)/e_{m}\left(z\right).$$

The relation between PL and the WCL is obtained after some manipulations [25] of Equations (6), (9), (11), (13) and (14):

(15) $$\begin{array}{c} S=S_{m}+S_{w},V=V_{m}-S_{w}\\ S_{w}=S_{c}ML_{w}S_{m} \end{array},$$

where $S_{c}$ = ${\left(1-\mathrm{MC}\right)}^{-1}$ is the sensitivity function of an ideal control loop that only includes M and C. From Equation (15), it is found that when $S_{c}\ll 1$, $S_{w}\ll S_{m}$, and the relation between PL and the WCL becomes:

(16) $$S\approx S_{m},V\approx V_{m}.$$

Equation (16) reveals a simplified procedure of the EMC design, since shaping $S_{m}$ and $V_{m}$ approximately shapes S and V when the bandwidth of the ideal control loop is much larger than that of the PL: $\omega_{BW,icl}\gg \omega_{BW,pl}$. Using this simplified procedure, the WCL shape is imposed by the PL shape within the frequency band of the ideal control loop, when the stopband of $S_{c}$ can be designed to be sufficiently wide with respect to $S_{m}$ and $V_{m}$. The tuning procedure of the state predictor loop shape performed by pole placement has been reported in detail in [26]. This procedure exploits the asymptotic equation of the loop shape and approximate relations between pole location and closed-loop transfer function, a method that demands complex manual calculation and some experiences [26]. In the following section, an improved design method of EMC is described, which could liberate control engineers from the tedious calculations and help them focus on modeling and in-field adjusting.

## 5. The Accelerometer EMC Tuning Using Non-Smooth Optimization

After building the main DCC structure in Figure 3, the tunable parameters include in total $m_{d}={\left[\begin{array}{ccc} m_{d0} & m_{d1} & m_{d2} \end{array}\right]}^{T}$, $l_{s}=\left[\begin{array}{ccc} l_{s0} & 0 & 0 \end{array}\right]$ and *β*: five parameters. As explained in the previous sections, the locations of the pole/eigenvalues of the state equation are the tie between the tunable parameters and the closed-loop transfer functions $S_{m}$ and $V_{m}$. In [18,26], several equations are given for computing the asymptotic loop shape of $S_{m}$ and $V_{m}$, but to the authors’ knowledge, the final accurate design is still that of trial-and-error and requires some practical experience.

On the other hand, algorithms for solving structured H-infinity optimization problems have been available in the control literature since 2005, based on the non-smooth optimization [27,28] whose numerical tools are integrated in a MATLAB toolbox [29]. In the classic H-infinity synthesis, controller design is usually generated by the algorithm itself during the calculation, the orders of which are the sum of the plant and weight functions. The non-smooth optimization technique makes it possible to design structured controllers that are inherited from other practical cases, including the EMC, without changing the controller software architecture [15]. This technique may utilize H-infinity theory for accurate transfer function design by avoiding the loss of performance or of the stability margin during the order reduction of non-structured H-infinity controllers.

In our case, the core of EMC design is to adjust the five tunable parameters to design the loop shape of $S_{m}$ and $V_{m}$. By applying the H-infinity theory with the help of the non-smooth optimization technique, the design procedure of EMC can be solved as a standard non-smooth optimization problem, which can be expressed in the following form:

(17) $$\begin{array}{c} \underset{K_{m}}{\mathrm{minimize}}\left(f\left(K_{m}\right)\right),f\left(K_{m}\right)={∥\begin{array}{c} W_{S}S_{m}\\ W_{V}V_{m} \end{array}∥}_{\infty }, \end{array}$$

where $K_{m}={\left[m_{d0}\;m_{d1}\;m_{d2}\;l_{s0}\;\beta \right]}^{T}$ is the assembly of tunable parameters, $W_{S}$ and $W_{V}$ are weighting functions of $S_{m}$ and $V_{m}$, that are the reciprocal of the design target of the $S_{m}$ and $V_{m}$ loop shape, and ${∥\cdot ∥}_{\infty }$ represents H-infinity norm. When the optimization algorithm reaches $f\left(K_{m}\right)\leq 1$, the singular values *σ* of $S_{m}$, $V_{m}$, $W_{S}^{-1}$ and $W_{V}^{-1}$ fulfill the following inequality:

(18) $$\begin{array}{c} \bar{\sigma }\left(S_{m}\right)\leq \bar{\sigma }\left(W_{S}^{-1}\right)\underset{SISO}{\Rightarrow }\left|S_{m}\right|\leq \left|W_{S}^{-1}\right|\\ \bar{\sigma }\left(V_{m}\right)\leq \bar{\sigma }\left(W_{V}^{-1}\right)\underset{SISO}{\Rightarrow }\left|V_{m}\right|\leq \left|W_{V}^{-1}\right| \end{array},$$

where $\bar{\sigma }$ represents the upper boundary of the singular value matrix and turns out to be a scalar magnitude frequency response in the SISO case. In this way, the target loop shapes of $S_{m}$ and $V_{m}$ can be bounded by $W_{S}^{-1}$ and $W_{V}^{-1}$.

Designing the magnitude of the sensitivity function and of the complementary sensitivity at the same time is called the “mixed sensitivity loop shaping” that is equivalent to the open-loop transfer function design. It is usually known as “open loop shaping” because of Equation (13) within the robust control theory. For the EMC state predictor design, in order to avoid some cases where the two functions $W_{S}$ and $W_{V}$ are designed to be incompatible with each other, the optimization target is re-decided by using a self-consistent open-loop shape function $W_{LS}^{N}$ (nominal loop target) for simplicity purposes, rather than setting $W_{S}$ and $W_{V}$ separately. Besides $W_{LS}^{N}$, a cross-frequency tolerance factor $\gamma_{fct}$ is applied to shift the $W_{LS}^{N}$ function curve to either lower or higher frequency and helps to obtain a pair of final loop shape boundaries $W_{LS}^{L}=\gamma_{fct}^{-1}W_{LS}^{N}$ and $W_{LS}^{H}=\gamma_{fct}W_{LS}^{N}$. Then, with a proper choice of $\gamma_{fct}$, the loop shape constraints will be equivalently transformed into the mixed sensitivity bounds as follows:

(19) $$\begin{array}{c} W_{V}=1+{\left(W_{LS}^{H}\right)}^{-1}=1+{\left(\gamma_{fct}W_{LS}^{N}\right)}^{-1},\;\\ W_{S}=1+W_{LS}^{L}=1+\gamma_{fct}^{-1}W_{LS}^{N}. \end{array}$$

**Figure 4:**
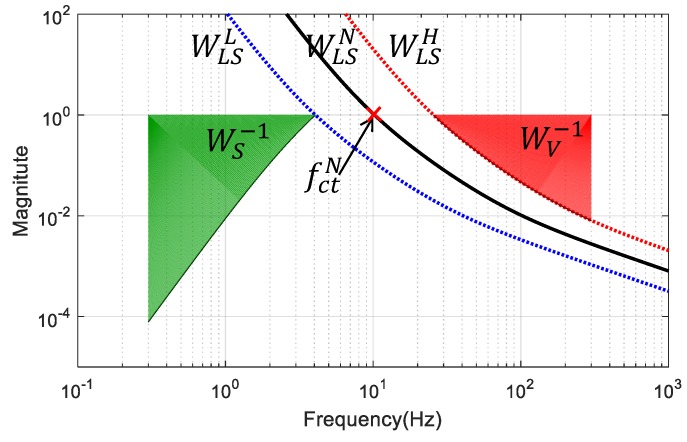
Loop shaping of the mixed sensitivity boundaries $W_{S}^{-1}$ and $W_{V}^{-1}$ by defining an open-loop transfer function $W_{LS}^{N}$ according to Equation (20) with a cross-over frequency $f_{ct}^{N}$ set as 10 Hz and $\gamma_{fct}=2.5$.

Here, a target loop shape, which could guaranty an 80-dB/dec disturbance rejection in the low-frequency domain, as well as at least up to −20-dB/dec roll-off in the high-frequency domain, is used:

(20) $$W_{LS}^{N}=\frac{0.1\omega_{ct}^{N}{\left(s+\omega_{ct}^{N}\right)}^{2}\left(s+10\omega_{ct}^{N}\right)}{s^{4}},$$

where $\omega_{ct}^{N}=2\pi f_{ct}^{N}$ is the target cross-over frequency. The calculation process of this final target loop shape is illustrated in Figure 4, in which the complementary sensitivity boundary $W_{V}^{-1}$ is obtained by the $W_{LS}^{H}$ function in the high-frequency range, and the sensitivity boundary $W_{S}^{-1}$ is obtained by the reciprocal $W_{LS}^{L}$ function in the low-frequency range. These two boundaries decide the green and red areas of Figure 4, which means that the resulting $S_{m}$ and $V_{m}$ should be constrained to stay outside of these forbidden areas according to Equation (18). The trade-off between disturbance rejection and robustness can be precisely adjusted by tuning $f_{ct}^{N}$ in Equation (20). The tolerance factor $\gamma_{fct}$ is empirically set to 2.5 here in order to leave enough space to the optimization routine for searching a compromise between low- and high-frequency performance, as well as for preventing transfer function overshoot.

**Figure 5:**
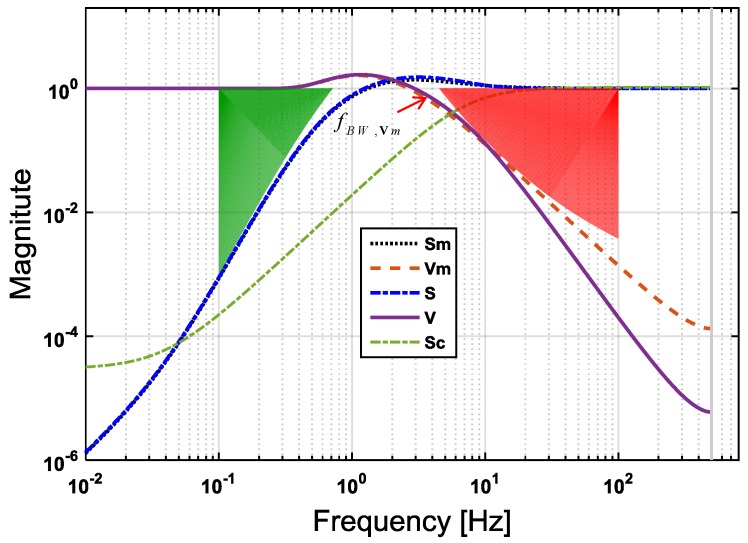
Closed-loop transfer functions as a result of the EMC of optimization when $f_{ct}^{N}$ = 2 Hz. The ideal function $S_{c}$ is also plotted for comparison.

**Figure 6:**
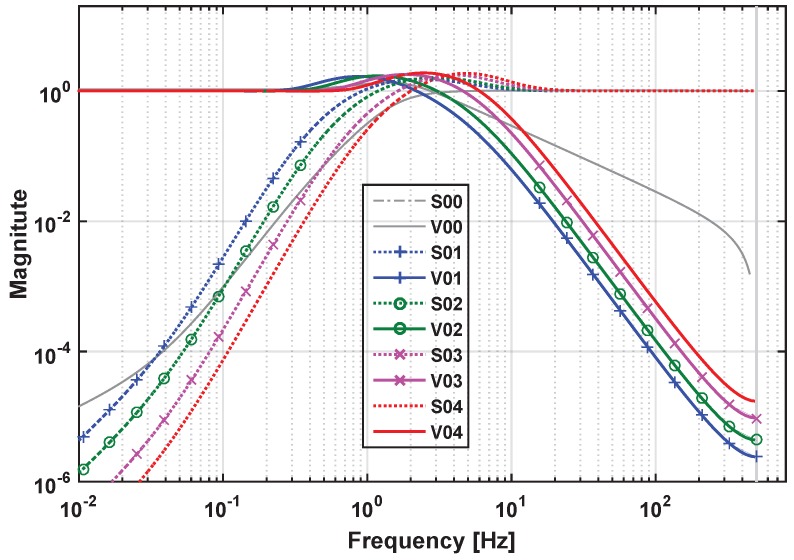
Series of closed-loop transfer functions as a result of the optimizations. Curves labeled “ctrl01” to “ctrl04” represent the results of the non-smooth EMC design with $f_{ct}^{N}$ equal to 1.5, 2, 3 and 4 Hz, respectively; the curve labeled “ctrl00” represents the result of a classical PID controller with a cross-over frequency of 3 Hz.

As explained in Section 4, the state feedback gains $\left[k_{p}\;k_{d}\right]$ could be tuned using simple PD feedback after choosing a sufficient wide control loop bandwidth. Here, $[k_{p}=-1136$, $k_{d}=-64]$ is set, which corresponds to a 10-Hz cross-over frequency of ideal control loop $S_{c}$. Figure 5 shows an example of how to optimize the loop shape for EMC state predictor design. In this figure, $f_{ct}^{N}$ is set to 2 Hz. After running the optimization routine, the resulting magnitude of the transfer functions, $S_{m}$ and $V_{m}$, as well as S and V, are clearly bounded by the colored forbidden areas in Figure 5. The discrepancy between $S_{m}$ and S, $V_{m}$ and V looks negligible in regions where the condition $S_{c}\ll 1$ is fulfilled. The overshoot of $S_{m}$ and $V_{m}$ is below 5 dB ensuring the adequate system stability margin. When $f_{ct}^{N}$ varies from 1.5 Hz–4 Hz, four designed $S_{m}$ and $V_{m}$ that are labeled ctrl01, ctrl02, ctrl03 and ctrl04 respectively, smoothly shift, as shown in Figure 6, and maintain the same slopes as the target functions. With the optimized parameters that are shown in Table 2 for these four controllers, their frequency performances are listed in Table 3. For the comparison, a classical PID controller that has the same cross-over frequency as ctrl03 is also plotted in Figure 6. Better disturbance rejection at 0.1 Hz can be observed by comparing the S curve of ctrl03 (EMC) with that of ctrl00 (PID, with parameters: $k_{p}$ = 119, $k_{i}$ = 191, $k_{d}$ = 18.3). From Table 3, it can be seen that the sensitivity $\left|S\right|$, also known as the disturbance rejection gain of ctrl03 at 0.1 Hz, is −75 dB, which is 14 dB better than that of ctrl00. This disturbance rejection strength increases with the decreasing of frequency and is found to be 34 dB better than that of ctrl00 at 0.01 Hz in this study case. The explanation is that in EMC, the second-order disturbance model acts as a double integrator giving a higher open-loop gain slope of up to 80 dB/dec. Moreover, the roll-off slope of the complementary sensitivity V in high frequency (20–500 Hz) is also better for ctrl03 than that for ctrl00 and, thus, improves the rejection performances of both the sensor noise and the high-frequency model uncertainties. Here, the bandwidth $f_{BW,Vm}$ in Table 3 is defined as the frequency where the magnitude of $V_{m}$ is equal to −3 dB, whose relative position is also pointed out in Figure 5 for a clearer demonstration.

Key design steps and the related MATLAB functions (in bold font) are listed for readers to reuse this work:

- Step 1: Build the Simulink model following Figure 3, where the signal names must be inserted;
- Step 2: Build the optimization target functions using TuningGoal.MaxLoopGain for $W_{V}$ and TuningGoal.MinLoopGain for $W_{S}$;
- Step 3: Use slTuner to define tunable parameters in the Simulink model;
- Step 4: Use systune to start optimization with output of Steps 2 and 3.

By continuously tuning $f_{ct}^{N}$ with a tiny step, e.g., 0.1 Hz, a lookup table of the corresponding optimized DCC parameters ($m_{d0}$, $m_{d1}$, $m_{d2}$, $l_{s0}$ and *β*) at different $f_{ct}^{N}$ in the same EMC controller structure can be generated. In this way, the researchers can focus on final tuning of the controller by looking up the table with new significant tunable parameter $f_{ct}^{N}$, which is closer to the physical meaning compared to $K_{P}$, $K_{I}$ and $K_{D}$ in PID tuning.

## 6. Simulation Results

**Figure 7:**
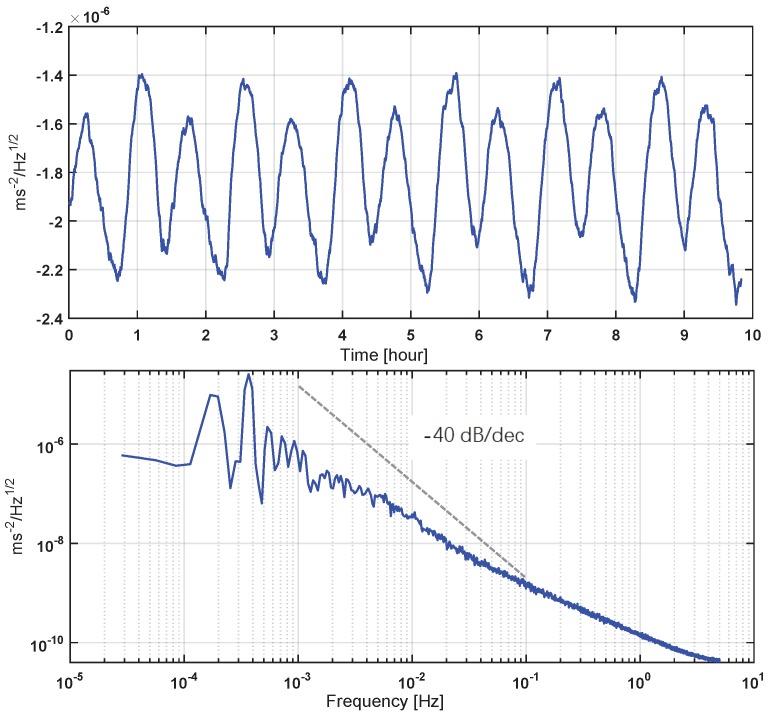
Input acceleration for closed-loop simulation in the time domain (upper diagram) and in the power spectral density (bottom diagram). A −40-dB/dec line is also plotted for comparison.

Simulated results were obtained from an end-to-end accelerometer numerical simulator developed in the MATLAB/Simulink environment, where the nonlinearity of the mechanical sensor head, the hardware transfer functions, as well as the noises are included. A segment of acceleration that is produced by orbit propagation and air-drag calculation of low-Earth-orbit satellites (like GRACE) with a decreasing PSD from 1 mHz to 5 Hz has been generated as the simulation input. This acceleration input is shown in Figure 7 in both the time and frequency domains, and its magnitude has been adjusted with respect to the instrument measurement range, with the peak input acceleration of about $2.5\times 10^{-6}$ $m/s^{2}$.

**Figure 8:**
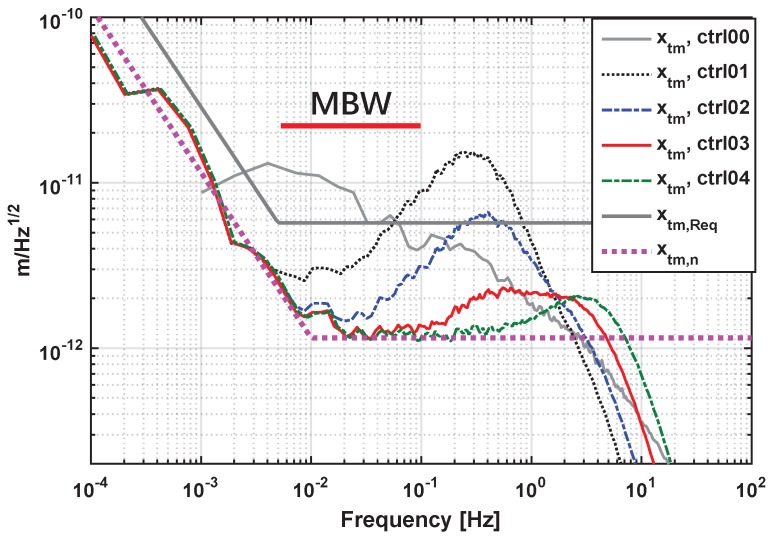
PSD of the residual TM relative position from the closed-loop simulations under four EMC designed controllers with $f_{ct}^{N}$ = 1.5, 2, 3 and 4 Hz: “ctrl01”–“ctrl04”; and a PID controller with a 3-Hz cross-over frequency: “ctrl00”. The measurement band (MBW) is also marked in the figure.

Simulation results of PSD of the TM position $x_{\mathrm{TM}}$, as the main performance criterion explained in Section 2, are shown in Figure 8. In this figure, the control performances of a PID controller, as well as four EMC controllers designed with different $f_{ct}^{N}$ are compared, whose transfer functions are plotted as “ctrl00” to “ctrl04” in Figure 6, with all of the possible noise sources considered. In this work, EMC controllers have been only compared with PID because PID is the most common and widely-used controller in various applications. Further performance comparisons of EMC with other more sophisticated controllers will be studied in future works. In Figure 8, it could be seen that the $x_{\mathrm{TM}}$ PSD performance of the EMC-designed controllers within the measurement band (MBW) is significantly improved with increasing $f_{ct}^{N}$. Controllers ctrl02–ctrl04 could meet the control design requirement defined by Equation (4) (grey solid line of Figure 8) within MBW. In comparison, the EMC controller “ctrl03” (with $f_{ct}^{N}$ = 3 Hz) shows a much better, almost five-times disturbance rejection of the input acceleration within MBW than the PID controller “ctrl00” that has the same 3-Hz cross-over frequency. This performance comparison allows us to select controller “ctrl03” or “ctrl04” (with $f_{ct}^{N}$ = 3 and 4 Hz, respectively) as the recommended controller for nominal science mode, with its PSD of residual $x_{\mathrm{TM}}$ reaching the position sensor noise limit (pink dashed line of Figure 8) in the frequency band lower than 0.1 Hz. Since the input acceleration is very conservative in the simulation, controllers designed with $f_{ct}^{N}$ from 2 Hz–4 Hz are acceptable and can be loaded for in-field switching.

**Figure 9:**
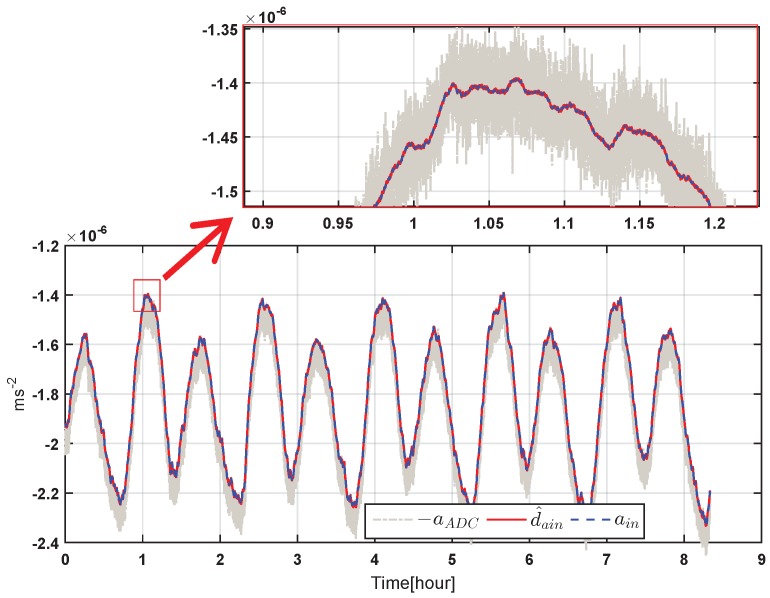
Simulated acceleration outputs of the EMC controller “ctrl03” at the feedback command port “$a_{ADC}$” and the disturbance predictor port “${\hat{d}}_{ain}$” in the time domain, along with the acceleration input $a_{in}$. Part of the curves are zoomed in the inset for clarity.

In an HPESA design, the acceleration signals to be measured are usually below 0.1 Hz. Therefore, for the sensor noise rejection consideration, the measurement bandwidth is preferred to be as low as possible; while on the disturbance rejection aspect, the control bandwidth should be as high as possible. In PID controllers, feedback command readout is the only measurement output; therefore, its measurement bandwidth is the same as the control bandwidth $f_{BW,V}$; while in this work, in the EMC framework, controllers could directly use the estimation of acceleration from the disturbance predictor model instead of the actual measurement; this is shown as ${\hat{d}}_{ain}$ in Figure 3. In this way, the measurement bandwidth $f_{BW,Vd}$ can be separated from the whole control loop bandwidth $f_{BW,V}$. Figure 9 shows an example of the simulated acceleration outputs’ comparison at different ports of the EMC controller in the time domain, where $a_{ADC}$ and ${\hat{d}}_{ain}$ are accelerations from the feedback command (shown in Figure 1) and from the disturbance predictor estimation, respectively. From the figure, it could be seen that the estimation of ${\hat{d}}_{ain}$ could precisely repeat the actual measurement of the acceleration input $a_{in}$, meanwhile preventing the disturbance of the high-frequency sensor noises, which contrarily have been introduced by the large control bandwidth at the output port of the feedback command $a_{ADC}$ that are plotted in the grey dashed-dotted line of Figure 9 for comparison. It should be pointed out that the noises in $a_{ADC}$ mainly come from the detection noises of capacitive position sensing, and the controller itself does not produce additional noises. These results show a good solution of the contradiction between enough measurement noise rejection and high control bandwidth by the EMC algorithms. This is one of the most significant advantages of EMC and is applicable to the inertial sensor-type accelerometers for the TianQin mission or other space missions that have even much lower measurement bands.

**Figure 10:**
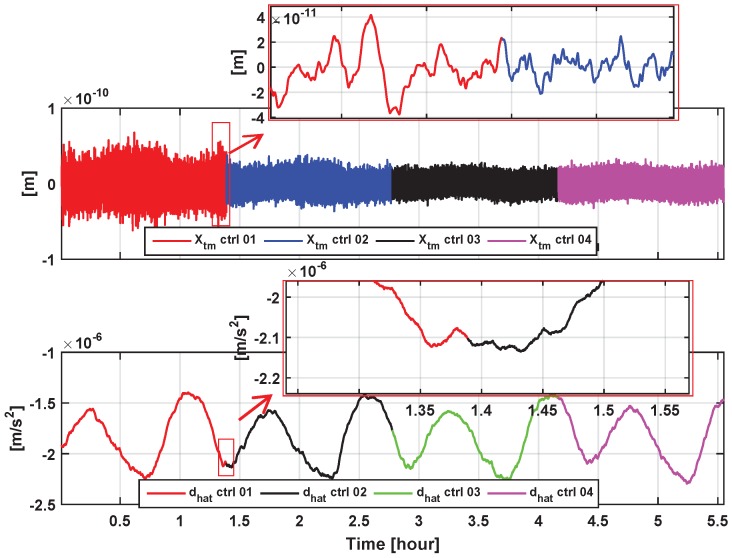
Simulated residual TM positions and accelerations under switches between four EMC control modes “ctrl01”–“ctrl04” in the time domain. Part of the curves are zoomed in the insets for clarity.

In a further simulation, the four EMC controllers “ctrl01”–“ctrl04” are sequentially switched on and loaded for about 1.5 h each. Figure 10 shows the simulation results of the residual TM positions and accelerations under smooth switches between the four control modes in the time domain. Smooth and seamless transitions of TM control can be observed during all of the switching instants, as shown in the TM accelerations, as well as the inset of the zoom-in positions of Figure 10. This may benefit more effective transitions between different working modes during in-orbit operations, e.g., the initial calibration mode and the scientific measurement mode, etc. The control mode switching could be implemented by using a remote-commanded multichannel switch for the preloaded parameters that are chosen. The implementation in the detailed FPGA controller codes will not be included here.

## 7. Conclusions and Outlook

In this work, a novel digital controller with disturbance observation and rejection is designed for the next generation HPESA of China. An improved design procedure of EMC controllers using non-smooth H-infinity optimization is demonstrated. The demo-designed controller ctrl03 provides a −80-dB disturbance rejection gain with only 4-Hz cross-over frequency. With this controller, the TM positions could be held tightly to the detection noise limit: 1.2 $\mathrm{pm}/{\mathrm{Hz}}^{1/2}$. This improved controller yields a better control performance of TM position, as well as a better integration of the control and data management functions for next generation HPESA, which have been verified by a series of end-to-end simulations. Novel functions of the improved EMC controller have been demonstrated in this work, including: (1) direct and accurate parametric design of the EMC loop shaping by simply encoding a lookup table according to the required bandwidth without complicated calculations; (2) predictions of all motion states for the data log; (3) better disturbance rejection performance than traditional controllers; (4) separation of the measurement bandwidth and the whole control loop bandwidth; (5) smooth switching between different controlling modes. Most of the functions described here are essential for the HPESA in gravitational wave detection missions, especially the combination of a high bandwidth disturbance rejection and a low measurement bandwidth, as well as the ability of smooth switching between different working modes of the controller. This design procedure and the non-smooth tuning method could be easily adopted to all kinds of servo-accelerometers [30,31], and the design results in this work will be tested in the next generation ultra-high resolution HPESA of China and also the core control unit of TianQin inertial sensors.

In this work, the EMC is designed by mainly focusing on the frequency performances of noise and lacks considerations of robust design against the neglected dynamics. This aspect of EMC has been carried out in several works using parameter scanning and optimization in recent years [25,32]. Further robustness analysis and design of the HPESA should be continued in the future with more details of plant hardware settled and tested.

## Figures and Tables

**Table 1:** Key parameters of the HPESA employed in our study.

Name	Symbol	Typical Value
Balance gap	$d_{0}$	200 μm
TM mass	$m_{\mathrm{TM}}$	320 g
Total balance capacitance	$C_{0}$	8.68 pF
TM bias voltage	$V_{b}$	4.5 V
Modulation voltage RMS	$V_{prms}$	5 V
Maximal feedback voltage	$V_{fed\;\max}$	2.5 V
Electrostatic stiffness	$\omega_{e}^{2}$	0.061 s${}^{-2}$
Position sensor noise at 0.1 Hz	$x_{n}$	$1.15\times 10^{-12}$ mHz${}^{-1/2}$
Feedback voltage noise	$V_{fed,n}$	$1\times 10^{-5}$ V/Hz${}^{1/2}$
Readout noise at 0.1 Hz	$V_{out,n}$	$0.5\times 10^{-6}$ V/Hz${}^{1/2}$
Measurement bandwidth	MBW	5 mHz–0.1 Hz
Digital control frequency	$T_{s}$	1 kHz

**Table 2:** Key parameters of the four EMC controllers with $f_{ct}^{N}$ equal to 1.5, 2, 3 and 4 Hz, respectively.

Controller	$m_{d0}$	$m_{d1}$	$m_{d2}$	$l_{s0}$	*β*
Ctrl01	−7.16	2.42	1.80	287	0.0292
Ctrl02	−17.5	7.86	7.86	518	0.0394
Ctrl03	−54.3	36.6	53.0	1120	0.0570
Ctrl04	−131	118	231	2010	0.0765

**Table 3:** Key control performances of four EMC and a PID controller. $f_{c}$: the designed cross-over frequency; $f_{BW,Vm}$: bandwidth of the predictor loop; $f_{BW,V}$: bandwidth of the whole control loop; $f_{BW,Vd}$: measurement bandwidth using ${\hat{d}}_{ain}$ (illustrated in Figure 3) as the output.

Controller	$f_{c}$ (Hz)	$f_{\mathrm{BW},Vm}$ (Hz)	$f_{\mathrm{BW},V}$ (Hz)	$f_{\mathrm{BW},Vd}$ (Hz)	${\left|S\right|}_{f=0.1\mathrm{Hz}}$ (dB)
PID: Ctrl00	3	N/A	4	N/A	−61
EMC: Ctrl01	1.5	2.64	2.93	0.66	−51
EMC: Ctrl02	2	3.53	3.93	0.90	−62
EMC: Ctrl03	3	5.29	5.88	1.31	−75
EMC: Ctrl04	4	7.14	7.93	1.76	−85
